# Crosstalk of Transcriptional Regulators of Adaptive Immune System and microRNAs: An Insight into Differentiation and Development

**DOI:** 10.3390/cells12040635

**Published:** 2023-02-16

**Authors:** Maryam Boshtam, Ilnaz Rahimmanesh, Laleh Shariati, Malihe Najaflu, Hossein Khanahmad, Mina Mirian, Atefeh Zarepour, Ali Zarrabi, Shirin Kouhpayeh

**Affiliations:** 1 Isfahan Cardiovascular Research Center, Cardiovascular Research Institute, Isfahan University of Medical Sciences, Isfahan 8187698191, Iran; 2 Applied Physiology Research Center, Cardiovascular Research Institute, Isfahan University of Medical Sciences, Isfahan 8187698191, Iran; 3 Department of Biomaterials, Nanotechnology and Tissue Engineering, School of Advanced Technologies in Medicine, Isfahan University of Medical Sciences, Isfahan 8187698191, Iran; 4 Biosensor Research Center, School of Advanced Technologies in Medicine, Isfahan University of Medical Sciences, Isfahan 8174533871, Iran; 5 Department of Genetics and Molecular Biology, School of Medicine, Isfahan University of Medical Sciences, Isfahan 8187698191, Iran; 6 Department of Pharmaceutical Biotechnology, School of Pharmacy and Pharmaceutical Science, Isfahan University of Medical Sciences, Isfahan 8187698191, Iran; 7 Department of Biomedical Engineering, Faculty of Engineering and Natural Sciences, Istinye University, Istanbul 34396, Turkey; 8 Department of Immunology, Erythron Genetics and Pathobiology Laboratory, Isfahan 7635181647, Iran

**Keywords:** miRNA, signaling pathway mediators, adaptive immune system, T-cells, B-cells

## Abstract

MicroRNAs (miRNAs), as small regulatory RNA molecules, are involved in gene expression at the post-transcriptional level. Hence, miRNAs contribute to gene regulation of various steps of different cell subsets’ differentiation, maturation, and activation. The adaptive immune system arm, which exhibits the most specific immune responses, is also modulated by miRNAs. The generation and maturation of various T-cell subsets concomitant with B-cells is under precise regulation of miRNAs which function directly on the hallmark genes of each cell subset or indirectly through regulation of signaling pathway mediators and/or transcription factors involved in this maturation journey. In this review, we first discussed the origination process of common lymphocyte progenitors from hematopoietic stem cells, which further differentiate into various T-cell subsets under strict regulation of miRNAs and transcription factors. Subsequently, the differentiation of B-cells from common lymphocyte progenitors in bone marrow and periphery were discussed in association with a network of miRNAs and transcription factors.

## 1. Introduction

The immune system in vertebrates is conventionally divided into innate and adaptive immunity [1,2]. Innate immunity comprises cells, which exert effects through recognition of patterns on invading microorganisms in a preprogrammed manner, whereas the adaptive immune system is conducted principally by lymphocytes through applying somatically rearranged genes to generate specific receptors for almost any epitopes which were neglected by the innate immune system. The adaptive defense system takes time to develop; nevertheless, it provides the host with eradication of infectious agents and long-lasting immunity [3].

**Figure 1:**
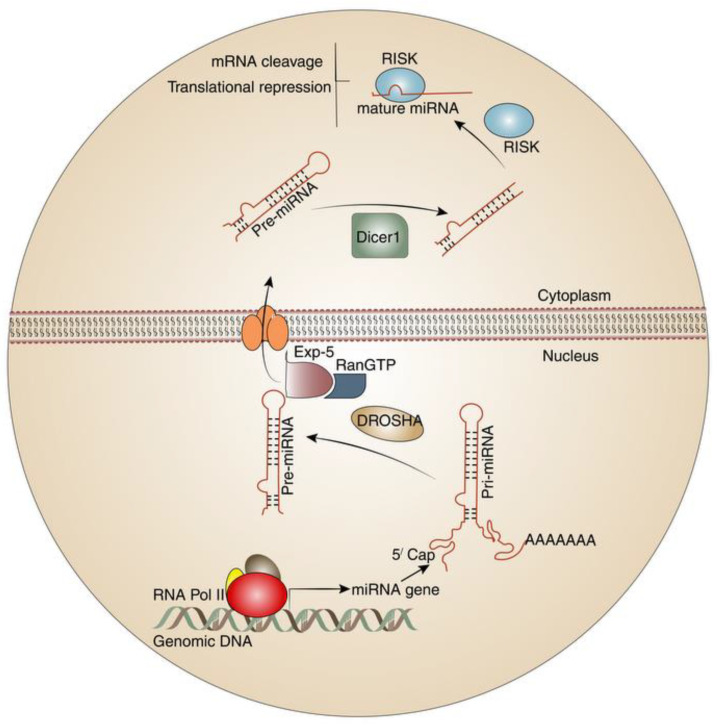
MiRNA biogenesis. Following transcription of miRNA by RNA polymerase in the nucleus, pre-miRNA was generated with a stem-loop structure. Immature miRNAs would be further processed by Drosha and Dicer in the nucleus and cytoplasm, respectively, leading to mature miRNA synthesis. The mature miRNA could incorporate into the RISC. The RISC complex binds to the target mRNA, leading to mRNA cleavage or translational regulation. Abbreviations: Exp-5: Exportin-5; RanGTP: GTP-bound Ran.

Molecular regulatory mechanisms of the adaptive immune system have been an emerging interest in the last years. One of the most inspiring parts of gene regulation has been discovered through the introduction of microRNAs (miRNAs). MiRNAs are introduced as small (19–22 nucleotides) non-coding RNA, transcribed by RNA polymerase II and rarely, RNA polymerase III [4,5,6]. Virtually 500–1000 miRNAs are expressed in human cells and their expression profiles vary in different organs and tissues. MiRNAs are firstly created as primary transcripts (pri-miRNAs) in the first step containing a stem-loop structure [7]. The primary transcripts are further processed by nuclear and cytoplasmic enzymes Drosha and Dicer, respectively, into mature miRNA that can be loaded into the RNA-induced silencing complex (RISC). Post-transcriptional gene regulation is mediated through binding of the RISC complex to the complementary sequence in the 3′ UTR of target mRNA and blocking translation [8,9]. The miRNA biogenesis is illustrated in Figure 1.

The role of miRNA in mammalian physiology was first documented through the aberrant expression of these elements in cancer cells. The function of miRNAs in the adaptive immune system was demonstrated with the selective expression of miR-181a in the thymus and miR-223 in the bone marrow. Moreover, several studies reported miRNAs to display a multidimensional regulatory effect in different developmental stages of the adaptive immune system, including hematopoietic stem cells (HSCs), differentiation into various cell lineages, and activation of innate and adaptive immune systems [10,11,12].

Two major elements of the adaptive immune system are T- and B-cells. T-cells are involved in the second-wave response of the immune system through engagement of the repertoire of T-cell receptors (TCR), and subsequently dictation of T-cell function, which could be regulated by miRNAs in each step of differentiation, development, and activation of T lymphocytes. While in the case of B-cells, there is a highly diverse repertoire of antibodies that are responsible for antigen recognition, generation, and maintenance of humoral immunity, and miRNAs are introduced as critical regulators of B-cells in each developmental stage [13].

According to the above-mentioned features, this review paper aims to highlight current findings on miRNAs’ role in the differentiation, development, and activation of T- and B-cells as major players in the adaptive immune system. For this purpose, we have reviewed and mentioned the most recent articles in the case of using miRNA for immune cell engineering.

## 2. MicroRNAs and T-Cells

The differentiation of HSCs into well-characterized T-cell subsets is a fine-tuned process in which various genes and microenvironments are required. MiRNAs, as one of the key controller non-coding RNAs, engage in regulating T lymphocytes’ fate. Thus, early in vitro and in vivo studies utilized broad miRNA depletion in T-cells by removing the key proteins associated with miRNA biogenesis [14].

### 2.1. Differentiation and Development

The differentiation and proliferation of blood cell reservoirs are originated from HSCs and are culminated in erythroid, myeloid, and lymphoid cell compartments. Based on the increasing number of studies, many miRNAs in this complex process have been reported, which are divided into three main groups: The first group includes miRNAs that contribute to regulating self-renewal/differentiation of the HSCs. MiR-29a seems to be the most important miRNA in the maintenance of stemness in HSC, while miR-125b accounts for the significant differentiation of miRNA in HSCs. The second group of miRNAs implicates reprogramming progenitor cells into myeloid or lymphoid lineages, among which miR-130 and miR-181 are the most significant miRNAs for commitment to myeloid and lymphoid lineages, respectively. The third category of miRNAs involves further differentiation of each cell subset. For instance, miR-155 is abundantly found in the T-cell lineage and drives more differentiation of this cell subset, while miR-17-92 is a key regulator of further differentiation in the B-cell lineage [15].

### 2.2. Common Lymphocyte Progenitors’ Differentiation into T-Cells

The T-cell subset is derived from common lymphocyte progenitors (CLP) in the bone marrow and will further mature in the thymus. T-cell commitment from CLP is dependent on Notch-1 signaling and involvement of GATA-binding protein 3 (GATA3) transcription factor (TF). In the first step of thymic progenitors’ differentiation into mature T-cells, β-chain gene segments, including V, D, and J, will rearrange to form pre-TCR for providing survival and proliferation signals. This checkpoint recruits various downstream proteins to be involved in the phosphoinositide 3-kinases (PI3K) signaling pathway [16].

Loss of function studies revealed the critical role of miR-181a1/b1 in thymocyte development by inhibition of regulators in the negative feedback loop of the Notch-1 signaling pathway [17]. Moreover, miR-181 was demonstrated to modulate phosphatase and tensin homolog (PTEN) in the PI3K pathway, while miR-181-deficient mice models give rise to severe defects in T-cell development [18,19]. PTEN is also a mutual target for various miRNAs, including miR-17, miR-19, and miR-21, which are differentially expressed in the period of T-cell development [20].

The T-cell maturation process is divided into three distinct stages based on the cell surface expression of CD4 and CD8 molecules on thymocytes. In the initial stage, thymic progenitors appear as double-negative (DN) cells (CD4-CD8-), while in the transitional stage, T-cells differentiate into double-positive (DP) cells (CD4+CD8+), and in the final developmental stage, T-cells will fully differentiate into single-positive (SP) cells (CD4+CD8-) or (CD4-CD8+) which migrate to peripheral lymphoid tissues [21].

Functional analysis of miRNA depletion via Lck-Cre-mediated excision of Dicer or Drosha in mice thymocytes revealed the most profound impact if achieved during the DN stage, and specifically with interference with Dicer rather than Drosha [22], while further differentiation past the DN stage remains intact [23]. Conditional deletion of Dicer presented to significantly reduce the cellularity of DP thymocytes with the greatest impact on αβ T-cells, while the number of γδ T-cells remains intact. Subsequently, the number of SP CD4+ or CD8+ peripheral T-cells and the global CD3+ T-cells reduced in the periphery [24].

Moreover, following glucocorticoid (GC)-induced stress in thymic involution, all the normal functions of the thymus are suppressed due to DP cell sensitivity to apoptosis [20,21]. One of the possible mechanisms in this process might be the global reduction in the miRNAs via downregulation of Dicer, Drosha, and DGCR8, and the blockage of mature miRNA generation [25].

T-cell factor 1 (TCF-1), known as a downstream target of Notch-1, subsequently induces GATA3 and B-cell lymphoma/leukemia 11b (Bcl11b), which are capable of induction of T-cell commitment after stimulation, even in the absence of Notch-1 signaling. In this regard, miR-181a was found to be implicated in the regulation of T-cell development through the activation of a Notch signaling pathway. MiR-181ab1-deficient mouse models revealed elevated amounts of negative regulators of a Notch signaling pathway, including Notch-regulated ankyrin-repeat protein and Numb [26].

The overexpression of miR-21 in early thymocytes and a significant decline in mature T-cells bring up a dynamic role for miR-21 in the regulation of intra-thymic T-cell development. To this end, an miR-21-deficient animal model was employed to evaluate the role of miR-21 in T-cell lineage commitment. The knockout study indicated the role of miR-21 in the promotion of an alternative lineage fate through regulation of Bcl11b in early DN thymocytes, while upregulation of Bcl11b is associated with a loss of miR-21 at the end of the DN stage of thymocytes’ differentiation [27].

Another miRNA involved in T-cell lymphopoiesis is let-7, which drives the unconventional T-cell subsets’ differentiation. The normal function of let-7 is diminished by Lin28 as a TF during fatal thymopoiesis, demonstrating the role of let-7 for an adult thymopoiesis [28].

E2A and myeloblastosis viral oncogene homolog (Myb) were found to be important TFs in Variable, Diversity, and Joining (V(D)J) recombination and β-chain expression, and act as a critical checkpoint in T-cell maturation and progression through the DN stage [29] which functions convergent with the Notch-1 signaling pathway [30]. The activity of E2A is negatively regulated by the inhibitor of DNA binding 3 (Ida3) TF upon induction of the survival signal in the late DN stage. During the V(D)J recombination, the reduced levels of miR-150 induce the overexpression of c-Myb, leading to β-chain rearrangement and further differentiation of thymocytes through the DN stage, survival and proliferation of DP cells, and ultimately, transition into SP thymocytes [31,32]. Another target of miR-150 in the T-cell lineage commitment process is Notch-3, and its role in T-cell development is poorly understood. Overexpression of miR-150 in vitro showed a negative regulatory effect on the survival and proliferation of T-cells through targeting Notch-3 [31]. Moreover, miR-16 was found to be involved in T-cell lineage commitment through the regulation of Myb, suggesting a prominent role for miR-16 in early T-cell development [33].

Proceeding of T-cell-committed cells from DN towards the DP stage is dependent on PU.1 as a hub TF encoded by the *Spi1* gene. *Spi1* was reported as driving the differentiation of T-cells by inducing proliferation, limiting access to non-T cell fate, and adjustment of timing in T-cell serial developmental stages.

Another TF which effectively controls T-cell differentiation is the RUNT-related transcription factor (Runx1), which is known as indispensable for early thymocyte development and expansion of β-chain-positive cells. Structural mutations in Runx1 lead to the restriction of further T-cell differentiation into late DN cells.

Overexpression of miR-146a in lymphoid progenitors of mice was shown to profoundly interfere with the normal thymopoiesis process, promoting T-cell proliferation and reducing the number of peripheral CD4+ T-cells [34,35]. An in vitro study indicated the role of ectopic miR-146a to positively regulate the expression of PU.1, GATA3, and to a lesser extent, Runx1 [36]. Although, this mechanism stimulates the activation of T-cells rather than differentiation and thymopoiesis. On the other hand, there are a number of miRNAs containing RUNX1-consensus sequences [37,38], which can positively or negatively regulate the generation of other target miRNAs relevant to thymopoiesis [39,40,41,42].

Bcl-11b, another downstream target of the Notch-1 signaling pathway, was identified as an ultimate TF for full commitment of early thymocytes in the DN stage. Bcl-11b knockout mice showed restricted T-cell development in the DN stage, while retroviral transduction of Bcl-11b into DN locked cells leads to progress in thymocytes development [30]. This process is strictly regulated by upregulation of miR-21 in the early DN stage towards downregulation of miR-21 in the late DN stage, which is associated with low and high levels of Bcl-11b, respectively [27]. Signal transducer and activator of transcriptions (STATs) family members have been introduced as pan-regulators of the T-cell differentiation and activation process. STAT5 stimulates T-cell development through upregulation of the *Mcl1* gene, which promotes DN cell survival and differentiation [43]. Expression of miR-155 was reported to promote proliferation of thymocytes through IL-2, STAT5, and IFN gamma signaling pathways through modulation of suppressor of cytokine signaling 1 (SOCS1) expression [44,45].

Using the miR-17-92 overexpressing mice model in the early DN stage, Xiao and colleagues showed the essential role of the miR-17-92 cluster for thymopoiesis, specifically for CD4+ T-cells [46]. In addition, employing a miR-17-92 knockout mice model revealed severe defects in the transition to the DP stage [47].

MiR-181 was also found to be enriched in the transitional stage of thymocytes and block Bcl-2, CD69, and TCR expression, which are responsible for the positive selection of lymphocytes and transition to the SP stage [21].

Following the expression of CD4 and CD8 molecules in the DP stage, early T-cells undergo a positive selection step by gauging the quality and quantity of the TCR:MHC/peptide complex interaction, which later results in SP cells (CD4+/CD8- or CD4-/CD8+) committed to helper or cytotoxic T-cell compartments based on recognizing MHC class II or MHC class I, respectively.

The ability of T-cells to discriminate self from non-self stems from the selection process provided by the thymus microenvironment in the DP stage of T-cell development. Although the recognition of self-peptide-MHC ligandomes by TCR expressed on DP thymocytes apparently elicits a lethal response, the classical affinity model of thymic selection proposes an appropriate concept for this paradox. Interaction of TCR and self-peptide-MHC complex provides essential signals for survival and commitment into SP cell lineage (positive selection), whereas the recognition of self-peptides with high affinity mediates a negative selection [48].

One of the conventional mechanisms employed in selection processes is apoptosis, in which miRNAs are introduced as a strong regulatory arm. The MiR-17-92 cluster was indicated to modulate Bcl-2-like protein 11 (Bim) and PTEN, which participate in cell apoptosis [49]. Transgenic mice with miR-17-92 cluster overexpression showed a hyperproliferation response concomitant with resistance to cell death, leading to lymphoproliferative disorders [1]. Moreover, miR-146a transgenic mouse models indicated an impaired positive selection and ultimately compromised central tolerance that led to an increase in the DP thymocytes and a reduction of SP CD4+ or CD8+ T-cells. Transcriptome analysis shed light on miR-146a to serve as a suppressor through direct targeting of genes, such as *il7a*, which are involved in signaling pathways that contribute to controlling apoptosis and/or survival in the positive selection process in the thymus [34].

**Figure 2:**
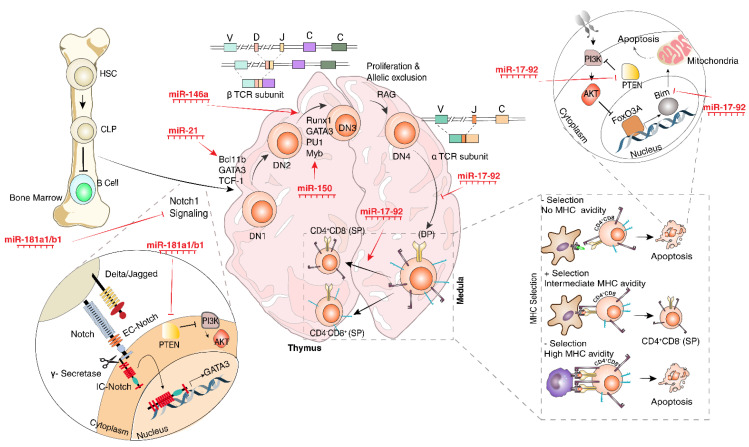
MiRNAs involved in common lymphocyte progenitor differentiation into T-cells. During the T-cell lymphopoiesis in the bone marrow and later in the thymus, various transcription factors and miRNAs are involved. This complex web of genes and proteins drives the V(D)J recombination through interaction with various transcription factors and signaling pathways, eventually mediating the single-positive (SP) T-cell generation from DN cells. Another set of miRNAs was reported to be involved in selection processes by inducing apoptosis or survival and proliferation. Abbreviations: FoxO3A: Forkhead box P3A; EC-Notch: Extracellular-Notch; IC-Notch: Intracellular-Notch.

The details of miRNAs’ role in T-cell subset generation from CLP, V(D)J recombination and selection processes are well-illustrated in Figure 2.

### 2.3. Cytotoxic T-Cells

CD4cre-mediated deletion of dcr-1 in thymocytes indicated an impaired progression from the DP to the SP differentiation stage, particularly for CD8+ single-positive cells [50]. The role of Dicer in T-cell thymic development cannot be addressed due to the insufficiency of Dicer excision before the DP stage and the preservation of mature miRNAs past Dicer ablation [51].

Cytotoxic T-cell lineage fate is dictated through the function of a transcriptional regulator, the Cd4 silencer, which is activated by Runx3 and represses Cd4 expression. Runx3 could also repress miR-181a-5p, which is a crucial regulatory element for governing CD4+ helper cell subset differentiation. Moreover, Runx complexes bind to the enhancer region of the Cd8 locus and will further drive the CD8+ T-cell differentiation [52,53].

Overexpression of miR-181a could also lead to a significant reduction in the number of CD8+ cytotoxic T-cells; however, the role of miR-181a in the T-cell developmental stage is still inconclusive [21]. Moreover, the high expression of miR-181a in the DP stage could be useful in the positive selection process through repression of the expression of Bcl-2, CD69, and TCR, which are required for positive selection [21], and represents the greatest impact on the transition to SP CD4+ T-cells. The miR-181a mechanism of action is based on the reduction in the TCR signaling threshold following modulatory function of phosphatase inhibitors such as Src homology region 2 domain-containing phosphatase-2 (SHP2) and protein-tyrosine phosphatase non-receptor type 22 (PTPN22), elevating phosphorylation of ERK and lymphocyte-specific tyrosine kinase [54].

Conversely, Th-inducing POZ-Kruppel factor (ThPOK), together with its homolog LRF, promotes helper CD4+ T-cell subset differentiation via repressing the Cd4 silencer, thus amplifying ThPOK in a positive feedback loop. The direct antagonistic interplay between ThPOK and Runx3 is now well-documented and known as the most important characteristic for determining T helper versus cytotoxic T-cell differentiation. Additionally, three different TFs, including MAZR, Gata3, and Myb, could interact with regulatory elements of the ThPOK locus to further drive MHC class II-restricted T lymphocytes [55].

In contrast to the miRNA network driving DP lymphocytes to T CD4+, the contributing miRNAs involved in proceeding CD4+CD8+ into CD4-CD8+ are relatively poorly understood. Ex vivo and in vivo studies illustrated miR-21, miR-155, and miR-146a assembly in a consistently upregulated manner in human primary CD8+ T-cells when compared to their naïve counterparts [56,57]. MiR-155 was determined to preferentially repress SOCS1, which negatively regulates common gamma chain cytokine signaling pathways in CD4+ mice T-cells and is proposed to play a crucial role in CD8+T cell expansion as well [58].

Contrarily, the comparative analysis showed that the miR17-92 cluster and miR-26a have a clear trend of reduction in differentiated T CD8+ cells [57]. Previous studies reported the traditional miRNA cluster role, miR-17-92, in T-cell differentiation [46], which was further categorized as one of the most expressed miRNAs in human CD8+ T-cells and correlated well with the degree of CD8+ T-cell maturation [57]. Although, the precise analysis of the miR-17-92 cluster revealed a differential intra-cluster regulation throughout CD8+ T-cell polarization and activation following antigen stimulation [57] with the maximum expression level during the expansion and proliferation of effector CD8+ T-cells [56]. The function of the miR-17-92 cluster is recommended to modulate Bim, and guarantee CD8+ T-cell survival and further expansion, which are still required to be validated [57].

Lie et al. demonstrated the role of miR-146a in the differentiation of CD8+ T-cells using a murine mouse model overexpressing miR-146a. This study revealed the role of miR-146a in the stimulation of a proliferative response in DP thymocytes, while limiting further differentiation into SP CD4+ or CD8+ T-cells, with a more severe effect on CD8+ T-cells [34].

Overexpression of miR-125b is associated with lymphoproliferative disorders with an increase in CD8+ expansion. This effect is mediated through remarkable overexpression of miR-125 in HSCs, which subsequently represses the pro-apoptotic targets, Bmf and KLF13, leading to uncontrolled T-cell-driven lymphoproliferation [59].

MiRNA profiling using microarray and quantitative PCR revealed that miR-142-3p and -5p are among the miRNAs with the highest expression in CD8+ T-cells. Although, miR-142-3p and miR-142-5p are known to be constitutionally involved in the T CD8+ cell subset rather than differentially, however, no definite transcriptional regulation has been elucidated for these two miRNAs, so far [57].

### 2.4. CD4+ T-Cell Fates

Following antigen exposure and based on the type of antigen and/or microenvironment characteristics, naïve CD4+ T-cells could be more designed into effector T-cell subsets, including Th1, Th2, Th17, T regulatory, and T-follicular helper (Tfh) subsets. Each of these cell lineages is characterized by distinct functions and a gene expression profile which is partially orchestrated with miRNAs [20].

#### 2.4.1. Th1 Cells

In the Th1 cell differentiation process, IFN-γ and IL-12 are first secreted by DCs and macrophages after encountering intracellular pathogens, which later activate STAT1 and STAT4, respectively. STAT1 induces T-bet as the hub TF in Th1 differentiation concomitant with nuclear factor of activated T-cell (NFAT), adapt or related protein complex 1 (AP-1), and nuclear factor jB (NFjB) following TCR involvement. On the other hand, STAT4 and T-bet act convergent to induce Runx3, which later mediates IFN-γ production in Th1 cells coordinated with T-bet in a positive feedback loop. Moreover, the STAT4, T-bet, and Runx3 axis would block Th2 programming by antagonizing Gata3 activity [30]. Additionally, Eomesodermin (Eomes), a TF of the T-bet family closely related to T-bet, drives the production of IFN-γ and further promotion of Th1 cell differentiation [60].

Although the role of miR-181a in cytotoxic T-cell subset differentiation was previously extensively investigated, the function of miR-181a in differentiation of the T helper cell subset still remains a controversy [10]. Previous studies have provided evidence on the role of Runx3 in the differentiation of T helper cells. Their data introduce the miR-181a-5p as the major regulatory agent in Th cell lineage differentiation, while it attaches to the Cd8 locus for further driving of CD8+ T-cell differentiation [10].

Genome-wide studies of potential candidate miRNAs revealed the top two hits, miR-29a and miR-29b, as the regulatory elements which suppress the production of IFN-γ directly while targeting T-bet indirectly, and thus inhibit Th1 cell development [61]. The mechanism of miR-29 action is illustrated by employing genome-wide target analysis of gene expression and repression following miR-29 transfection and inhibition, respectively. MiR-29 was shown to repress T-Box transcription factor 21 (Tbx21) and Eomes as fundamental TFs driving Th1 cell differentiation, and thus the miR-29 expression level was found to be the lowest among all T helper cell subsets. Transgenic mice overexpressing miR-29 binding sites were employed for evaluation of the sponge effect in competition with endogenous miR-29, which demonstrated the increased IFN-γ production; whereas, miR-29-deficient T-cells were found to overexpress TBET and IFN-γ [62]. The underlying mechanism by which miR-29 is downregulated in the Th1 cell subset is occupying the miR-29 promoter by NF-κB. On the other hand, miR-29 is illustrated to be induced by IFN-γ in a negative feedback loop [63]. Furthermore, the miR-17-92 cluster displayed a dual function in T-cell proliferation through targeting PTEN and the pro-apoptotic protein Bim and IFN-γ production [64]. Refueling of miR-17-92-deficient T-cells with each of the miRNAs in this cluster revealed that miR-19b is exclusively capable of promoting IFN-γ production and T-cell proliferation, while miR-19a reconstitution was incompetent to exert T-cell proliferative function. Additionally, miR-17 along with miR-19b were presented to reduce the activation-induced cell death in miR-17-92-deficient T-cells, whereas the introduction of miR-18a interestingly inhibited proliferation and induced apoptosis. MiR-17-92 cluster deficiency is associated with a disturbed DTH response and impaired tumor rejection, emphasizing the role of the miR-17-92 cluster in Th1 differentiation and response. Furthermore, the miR-17-92 cluster is involved in the production of cytokines, including IFN-γ, IL-2, IL-4, IL-5, and TNFα, intriguingly, which proposes the supportive role of miR-17-92 in multiple T helper and probably in CD8+ T-cell subsets [43].

As discussed earlier, miR-146a was found to have a strong immunoregulatory effect on thymocytes and T-cell subsets, with a more severe effect on CD8+ T-cells [34,65]. MiR-146a suppresses the Th1 cell differentiation by targeting STAT1 [44]. Further activation of T-cells leads to NF-κB-mediated induction of miR-146a, which targets TNF receptor-associated factor 6 (TRAF6) and interleukin-1 receptor-associated kinase 1 (IRAK1) and forms a negative feedback loop for governing NF-κB signaling [65]. Moreover, in vivo and in vitro studies revealed significantly reduced IFN-γ production in miR-146a-deficient CD4+ T-cells [44,65,66].

In contrast to miR-146a, miR-155 was shown to enhance Th1-mediated tissue inflammation [67] through repression of negative regulators in cytokine signaling, such as SOCS1 and SH2 domain-containing inositol polyphosphate 5-phosphatase 1 (SHIP1) [66,68]. In addition to the Th1 cell response, miR-155 might be responsible for the interruption of CD4+ T-cells’ differentiation into the Th1 subset through targeting IFN-γ receptor mRNA and ultimately, downregulation of IFN-γ [69].

In vivo experiments using miR-146a and miR-155 dual-deficient mice models provided evidence of the dominant role of miR-155 over miR-146a, while presenting an opposing role [66]. MiR-155-deficient mice models are resistant against experimental autoimmune encephalomyelitis (EAE), accompanied with a lower number of lymphoid cell infiltration [67].

There are also miRNAs (such as miR-21 and miR-24) that affect Th1 cell differentiation via interaction with immune system elements, and therefore are indirectly involved in the differentiation process. Previous experiments clearly stated the role of miR-21 in the negative regulation of IL-12 production from DCs and thus indirectly targeted the T-bet, which is responsible for Th1 subset differentiation [70]. In addition, miR-24 was found to participate in IFN-γ and IL-17 generation through suppression of TCF-1 in corresponding T-cell subsets, which is in contrast to the previously reported miR-24 role in driving Th2 responses [71]. TCF-1 is capable of GATA3 induction through the interaction with b-catenin and further promotion of Th2 cells. Although, restoration of TCF-1 expression in a transgenic mice model overexpressing miR-24 showed downregulation of GATA3 and IL-4, which is in favor of Th2 cell differentiation [72].

#### 2.4.2. Th2 Cells

Exposure of naïve T-cells to extracellular pathogens such as parasites committed these cells for further differentiation towards the Th2 cell subset through the involvement of IL-4R and activation of STAT6, in corporation with NFjB, AP-1, and NFAT. Taken together, these signals lead to the amplification of Gata3 as the Th2 lineage-defining TF. Gata3 leads to its overexpression in a positive feedback loop while simultaneously driving the expression of other genes, including interleukin-4 (IL-4), IL-5, and c-musculoaponeurotic fibrosarcoma (c-Maf) TF, of which the latter helps to induce further expression of IL-4. Furthermore, STAT5 is also found to play a role in the expression of TCR-induced IL-4Rα [54]. Another function attributable to STAT5 is driving NLRP3 expression, which further forms a complex with interferon regulatory factor 4 (IRF4) to induce Th2 cytokines, including IL-4, IL-5, and IL-13 [73].

Although much less is known about the central role of miRNAs in the regulation of Th2 differentiation and further response-facing antigens when compared to Th1 cells, there are still experiments reporting miRNAs’ expression profiles through Th2 differentiation. For instance, an in vitro study demonstrated that miR-155-deficient T-cells display impaired Th2 cell differentiation through targeting c-Maf as one of the major Th2 cells promoting TF [74,75]. The c-Maf, an IL-4 promoter transactivator, is the target of miR-155, which was first identified as a negative regulator of Th2 commitment. In addition to c-Maf, the possible suppression of Th2 development was linked to a depression of PU.1, which controls GATA3 expression [75,76]. Similarly, Okoye et al. indicated that mice with miR-155/T-cells had less airway disease due to miR-155’s role in reducing the expression of sphingosine-1-phosphate receptor 1 (S1P1), which is necessary for lymphocyte egress from lymphoid organs [77]. Moreover, employing miR-155-deficient mice models revealed failed production of IL-13 and IL-5 in the Th2 response context [78].

Let-7 cluster members were found to directly target IL-13 in vitro and control the Th2 cell response [79,80]. Moreover, Let-7 downregulation is well-associated with increased IL-10 expression in HIV patients, showing the regulatory effect of Let-7 on IL-10 mRNA in the Th2 cell subset [81].

Ectopic miR-21 overexpression polarizes T-cell differentiation into the Th2 cell subset through increasing GATA3 expression in vitro [82,83]. Furthermore, previous experiments provided a large body of evidence to define miR-21 as a major target for BCL-6, which is downregulated through binding of BCL6 to the corresponding promoter [84]. A molecular study indicated the role of miR-21 in the promotion of the Th2 cell response through repressing IL-12 secretion [44,85]. Recent findings revealed higher serum levels of miR-21 in asthmatic patients in comparison with healthy controls, and also a positive correlation between miR-21 and IL-4 amounts in asthmatic patients [60].

Overexpression of miR-27 or miR-128 decreases the naïve CD4+ T-cells and inhibits the Th2 anti-inflammatory response, favoring the Th1 cell response in multiple sclerosis patients. The regulatory effects of miR-27 and miR-128 on Th2 cells are mediated through targeting GATA3 and subsequent inhibition of IL-4 expression, leading to a Th2 to Th1 cytokine shift. Adoptive transfer of miR-27- and miR-128-transfected cells to EAE models restored the Th1 lineage function, leading to the exacerbation of encephalomyelitis [83].

In vitro studies revealed that the pharmacological inhibition of miR-182 slightly decreased the proliferation of Th2 cells. Moreover, adaptive transfer of cells treated with miR-182 antagomirs led to milder arthritis in vivo [86]. The expression level of miR-182 increased in Th2 cells, which is proposed to be due to the involvement of TCR in parallel with IL-2. On the other hand, miR-182 as a target for STAT5 is upregulated after miR-182 binding to the STAT5 locus, which is the possible explanation for high expression levels of miR-182 in Th2 cells [43]. Induction of miR-182 negatively regulates FOXO1 expression and ultimately controls the proliferation of Th2 cells in the phase of clonal expansion and IL-9 production from Th2 cells [87,88]. In addition, FOXO1 might play a role in Th2 cytokine production via binding and transactivation of the IRF4 promoter [87]. Although, miR-182 transgenic or knockout animal models are required for a better evaluation of miR-182’s role in the differentiation of Th2 or other Th cell subsets in vivo.

In the phase of the Th2 response, miR-17-92 transgenic mice showed increased expansion, particularly of CD4+ T-cells due to suppression of Bim and PTEN as major contributors to cell apoptosis [10]. Moreover, miR-17-92 knockout CD4+ T-cells revealed profoundly reduced production of IL-4, IL-5, and IL-13, in contrast to miR-17-92 transgenic CD4+ T-cells in vivo. The cytokine production deficiency in miR-17-92 knockout CD4+ T-cells was reconstituted following transfection of miR-19a and miR-19b mimics into these cells. Functional screening of miRNAs’ target genes revealed Pten and Socs1 as miR-19 target genes which negatively regulate Th2 lineage cytokine production [89]. Another miRNA, which is involved in Th2 cell differentiation and activation, is miR-126. In spite of Th2 cell constitution in immunity against allergic disease, employment of miR-126 antagomirs in animal models of allergic airway disease revealed a poor Th2 cell response [23]. The miR-126-mediated effect is exerted through upregulation of B-cell oct-binding factor-1 (OBF.1/BOB.1) and inactivation of PU.1, and thus removes negative regulation of GATA3 as the major Th2 cell-defining TF [50].

#### 2.4.3. Th17 Cells

Th17 plays a major role in pathogenesis of autoimmune diseases and defense against extracellular pathogens. The Th17 generation from naïve CD4+ cells is under stimulation of a complex web of TGF-β, IL-6, and IL-23. Th17 cell lineage is known for the expression of IL-17 and retinoic acid receptor-related orphan receptor-γt (RORγt). Following IL-6 and IL-23 secretion, STAT3 is activated, which functions coordinately with RORγt to synthesize IL-17 as the hallmark of the Th17 cell subset. Moreover, SMAD2 recruited by TGF-β and NF-kB functions parallel with ROR-γt to express Th17-specific genes.

Dicer-deficient mice models showed bolstering Th17 cell responses due to diminished Treg function, although these data are inconsistent with those of other in vitro studies [20]. The Th17-specific miRNA expression signature indicated miR-326 as a major contributor in Th17 effector cell differentiation, expanding Th17 cell numbers. Evaluation of the miR-326 expression level in Th17 cells of the EAE model positively correlates with disease severity; however, in vivo study results conflict [20]. The role of miR-326 is targeting ETS1 as the negative regulator of Th17 cell differentiation, thus enhancing IL-17-secreting cell generation from naïve CD4+ T-cells. However, there is still a discrepancy between in vivo and in vitro studies and numerous challenges are yet to be addressed to identify the role of miR-326 in Th17 cell polarization [43].

Relatively recent studies engage miR-301a for Th17-specific gene regulation since miR-301a is highly expressed in murine Th17 cell subsets. Inhibition studies using antagomirs illustrated a positive contribution role of miR-301a in Th17 cell differentiation through suppression of protein inhibitor of activated STAT 3 (PIAS3), as a negative regulator of the IL-6/IL-23/STAT3 axis. On the other hand, miR-301a mimics the treatment of CD4+ T-cells and exacerbates the EAE symptoms through increased CCR6 expression [20,50].

Expression of miR-155 seems to contribute to tissue inflammation through direct and indirect mechanisms of action regulating Th17 differentiation. Recent in vitro and in vivo studies evidenced the intrinsic role of miR-155 in the enhancement of STAT signaling pathways through suppression of SOCS1 and thus inhibition of Th17 cell subset origination from T CD4+. On the other hand, the indirect effect of miR-155 on Th17 cell differentiation is the suppression of Th17 cell-driving cytokine secretion by the DCs. The role of miR-155 in Th17 differentiation has been approved in helicobacter pylori, colitis, and EAE mice models [20,50].

Another Th17 polarizing miRNA is miR-182, which is overexpressed in Th17 cells after IL-2 signaling. It has been proposed that STAT5 signaling following IL-2 stimulation induces miR-182, which represses STAT5 in return in a negative feedback loop. MiR-182’s role in Th17 differentiation is exerted through repressing Foxo1 expression. Although, the results of in vitro studies using antagomirs are significantly different from what has been observed in those with miRNA ablation [43].

In the differentiation process of Th17, miR-10a plays a major role to balance Th17 and Th1, with a mutual interaction of T-bet. It is now known that miR-10a favors Th1 differentiation in a retinoic acid-rich microenvironment, which is the inducer of miR-10a and T-bet expression. MiR-10a directly targets Bcl-6 mRNA, resulting in the downregulation of Bcl-6 and thus increased Th17 cell differentiation in a T-bet-dependent pathway [90]. Previous studies have provided evidence for the role of Bcl-6 as a negative regulator in Th17 cell differentiation. Employment of RNAi showed repression of Th17 cell subset differentiation by inhibition of RORγt expression. It seems the administration of RNAi targets Bcl6, which in turn limits the function of T-bet as the inhibitory factor of RORγt expression [91].

Another confirmatory study underlined the role of miR-10a in Th17 differentiation through overexpression of miR-10a in MOG-specific CD4+ T-cells and postponing the MS course in the EAE model [20].

#### 2.4.4. Treg Cells

Treg cells are defined as having the ability to suppress immune system responses and T-cell homeostasis maintenance with the expression of factor forkhead box P (FOXP) 3 as the hallmark TF. Tregs are originated from the thymus or differentiated from naïve CD4+ cells in the periphery. Induction of FOXP3 contributed to the function of the complex network of TFs and cytokines such as TGF-β and IL-2. TFs, including NF-kB (c-rel), Foxo1, Foxo3, SMAD2, NFAT, AP-1, STAT3, STAT5, NR4a1, and Satb1, form a complex regulatory network in response to secreted cytokines, to induce or suppress FOXP3.

Selective deletion of Dicer or Drosha in Foxp3+ Tregs causes a loss of tolerance and induces autoimmunity, which shows the significance of miRNAs to restrain the tolerogenic effect of Tregs.

A network of miRNAs, including miR-320, miR-146a, miR-130a, miR-99a, miR-126, and miR-31, was found to govern Treg differentiation. A large body of research demonstrated the role of miR-155 in thymic Treg (tTreg) or natural Treg (nTreg) induction without affecting Treg function using miR-155-deficient mouse models. A positive feedback loop exists between miR-155 and Foxp3, in which differentiated Foxp3+CD4+ cells induce miR-155 expression, which reciprocally enhances the Foxp3 expression level and stability. In the derivation process of Treg from naïve T-cells, the IL-2R signaling pathway is stimulated via recruiting STAT5, which is diminished in a miR-155 knockout mice model. In vivo studies revealed that miR-155 functions through STAT5 downregulation while upregulating SOCS1, a negative regulator of STAT5 signaling. Furthermore, a C-type lectin-related pathway was found to modulate thymic generation of Treg cells through the expression of miR-15, proposing the complex role of miR-155 in thymic Treg generation [92].

High expression of miR-21 has been observed in Tregs, which plays a key role in negatively regulating Foxp3 expression. Moreover, reduced expression of miR-21 was reported as being associated with autoimmune states such as rheumatoid arthritis. In vitro studies using PNA suggested that miR-21 induces homeostasis of Tregs through modulating STAT3 and STAT5 in the IL-2R signaling pathway [93]. In conclusion, although STAT3 and Ap-1 are the inducers of miR-21, BCL-6 was found to negatively regulate miR-21 through the STAT3 binding element [82].

Based on the silencing effect of miR-29ab on IFN-γ production in Tconv cells, transgenic and knockout animal models are still required for a better evaluation of miR-29ab’s role in the promotion or inhibition of IFN-γ producing Tregs’ differentiation [20].

Additionally, miR-155 concomitant with miR-124a involves in iTreg generation through induction of Foxp3 expression after repression of histone deacetylase, sirtuin-1 [93].

Screening studies introduced a network of miRNAs involved in periphery-derived Tregs or induced Treg (iTreg) differentiation. One of the major negative regulators of iTreg generation is the mammalian target of rapamycin (mTor), which is strictly regulated by the miRNA web. For instance, miR-150 blocks mTOR and thus triggers iTreg differentiation, and administration of the miR-150 antagomir was shown to reduce iTreg generation, although miR-150-mediated iTreg generation occurs only in the presence of miR-99a. Other in vivo and in vitro studies revealed the other cooperating miRNAs in iTreg subset differentiation, miR15a/16 and miR15b/16, which target the components of the mTOR signaling pathway [94].

There is a controversial role assigned to miR-10a in Treg generation. Ablation of miR-10a was shown to be dispensable for Treg differentiation and for Treg function in autoimmune mice models. Likewise, downregulation of Foxp3 expression was observed in miR-10a-deficient mice, although the application of miR-10a antagomirs destabilized Foxp3 expression in vitro. This discrepancy might be due to the off-target effects of miR-10a and the compensative function of miR-10b in vivo. Another in vitro study showed that miR-10a makes iTregs capable to convert to Tfh through directly repressing BCL-6 [43].

Overexpression of miR-100 in CD4+ T-cells was evidenced to inhibit iTreg differentiation via repressing the mTor pathway. Interestingly, applying a manipulated miR-100 (C to U transition) culminates in TGF-β signaling enhancement and thus iTreg generation by changing the miR-100 target from mTor to SMAD2. Another miRNA targeting mTor pathways is miR-126, which inhibits the PI3K-AKT pathway in Tregs as well. The PI3K activation upregulates AKT, leading to Foxp3 augmentation and finally iTreg differentiation. The role of the miR-17-92 cluster in iTreg differentiation has also been reported, which is based on CD28-induced costimulatory signals. It has been proposed that miR-17 directly targets TGF-βR II and TGF-β signaling pathway components, implicating it in iTreg generation. On the other hand, in vitro studies provided enough evidence to reveal the critical role of CD28 signals in repressing Foxp3 expression in conventional T-cells through induction of the miR-17-92 cluster [43].

However, similar to what was observed earlier in miRNAs’ ablation in Tregs, in vitro studies illustrated no changes in Treg number or function subsequent to miR-17-92 cluster ablation in T-cells or Tregs. Another miRNA cluster with a regulatory/effector outcome on iTreg is the miR-23-27-24 cluster, in which miR-23 and miR-27 negatively regulate the differentiation of iTregs while miR-24 promotes iTreg cell subset development. A previous study illustrated miR-24 as the only member of the miR-23-27-24 cluster which actively targets Smad7 as the negative regulator of the TGF-β signaling pathway and thus promotes TGF-β-induced iTreg generation [95].

MiR-31 is illustrated to be overexpressed following TCR signaling induction, which negatively regulates iTreg cell differentiation. The direct target of miR-31 was found to be Gprc5a, known as retinoic acid-inducible protein 3, which further stimulates pTreg generation [96].

Collectively, these data suggest an antagonistic role for miRNAs, including miR-155, miR-21, miR-124a, miR-150, miR-99a, miR15a/16, miR15b/16, miR-10a, miR-17-92, and miR-24, while the other set of miRNAs, such as miR-100, miR-126, miR-23, miR-27, and miR-31, are agonistically effective on Tregs’ functions to induce Foxp3+, B lymphocyte-induced maturation protein-1 (BLIMP-1), and IL-10-secreting Tregs.

#### 2.4.5. Tfh Cells

T-follicular helper (Tfh) is a distinct T CD4+ cell subset that is specialized in helping B-cells to generate humoral immune responses and is characterized by the expression of IL-21, BCL-6, IL-10, and inducible T-cell co-stimulator (ICOS) [97]. Differentiation of Tfh is regulated by cytokines and TFs common with Treg, including IL-6 and STAT3, although the master regulator of Tfh is Bcl6, which acts simultaneously with IRF4, STAT5, c-Maf, and basic leucine zipper ATF-like Transcription Factor (Batf). ICOS and IRF4 act convergently to produce IL-10 as the hallmark cytokine of Tfh [98,99,100,101].

Expression profiling revealed a similar set of miRNAs enriched in both Tfh and Tregs, while functioning distinctly in follicular versus Tregs [43]. Substantial evidence demonstrated the role of the miR-17-92 cluster in Tfh generation and germinal center (GC) formation [102]. The consequence of miR-17-92 ablation is to reduce the Tfh cell number and the antibody response, although the defect was resolved using adoptive T-cell transfer and immunogen [103]. In addition, overexpression of miR-17-92 with CD4cre in T-cells triggered Tfh differentiation in Peyer’s patches as the site of stimulation for GC formation [104]. As described above for Tregs, miR-17-92 targets and represses PTEN, which is known as an inhibitor of Tfh cell generation. Moreover, miR-17-92 could act as a negative regulator of Tfh through inhibition of BCL-6, which is responsible for the repression of hub TFs in Th1, Th2, and Th17 [103]. Thus, miR-17-92 functions as a positive or a negative regulator of Tfh in a time-dependent manner.

MiR-155 was shown to be involved in chronic inflammation and induction of Tfh differentiation, while miR-146a negatively regulates Tfh generation through inhibition of ICOS expression [105].

**Figure 3:**
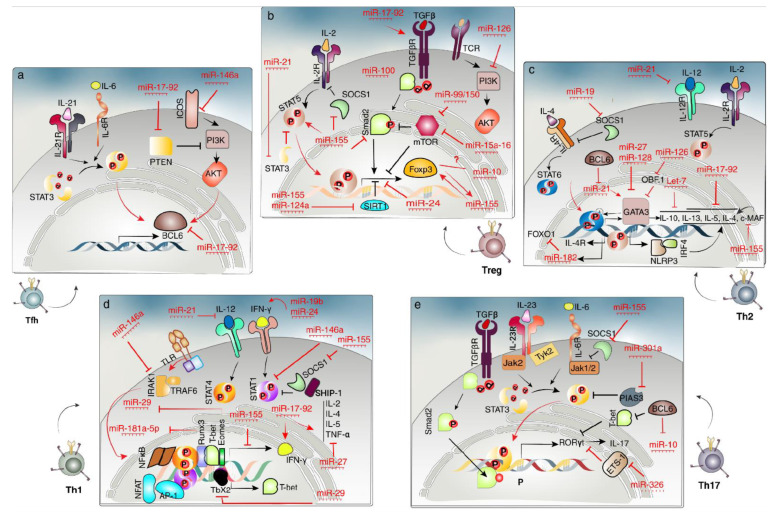
Schematic of the miRNAs’ interaction with transcription factors, signaling pathways, and genes, which are prominent in Tfh (**a**), Treg (**b**), Th2 (**c**), Th1 (**d**), and Th17 (**e**) cell subsets’ differentiation. Abbreviations: TLR: Toll-like receptor; Jak 1/2: Janus Kinase 1/2; TYK2: Tyrosine Kinase 2; ETS-1: ETS Proto-Oncogene 1, Transcription Factor.

Figure 3 summarizes the differentiation of various T-cell subsets regarding the involved TFs and miRNAs.

## 3. MicroRNAs and B-Cells

As described earlier for T-cells, B-cell commitment from HSCs is also orchestrated by a network of TFs and molecular mechanisms in the bone marrow and peripheral lymphoid organs. The cloning and sequencing studies led to the identification of miRNAs that are uniquely expressed in distinct cell types or developmental stages.

### 3.1. CLP Differentiation into B-Cells

As described earlier, the differentiation of HSCs into lymphocytes is facilitated through stromal signals in bone marrow, allowing HSCs to differentiate into CLPs in the first step of fine-tuned and stepwise processes of cell subsets’ differentiation. The expression of c-kit and IL-7 receptors is responsible for providing survival and proliferation signals for CLPs and further differentiation into lymphocytes [106].

The differentiation of CLPs into pro-B-cells is dependent on the E2A, early B-cell factor (ECF), IKAROS, and Paired Box 5 (PAX5) as major TFs in B-cell differentiation of both human and mice [106,107,108,109,110,111]. Bone marrow development of pro-B-cells initiates with a highly ordered sequential rearrangement of V, D, and J segments upon overexpression of recombination-activating (RAG) enzymes to generate IgM-expressing immature B-cells [112]. At this point, some immature B-cells develop into autoreactive lymphocytes due to recognizing self-antigens with high affinity, which leads to further V(D)J recombination in the light-chain or immature B-cell apoptosis that are together known as central tolerance. Another mechanism of tolerance is exerted in peripheral organs through receptor editing of self-reactive B-cells and is known as peripheral tolerance [113,114,115]. Immature B-cells migrate to the spleen for further maturation [112], and ultimately, mature B-cells with IgM and IgD co-expression were generated, which would be activated following antigen exposure [116].

The role of miRNAs in the regulation of B-cell development was first characterized through functional analysis of miRNA ablation in B-cells of mice models via Mbl-Cre-mediated Dicer excision, leading to complete blockade of pro-B-cells as well as inhibition of pro- to pre-B-cell transition [117]. Furthermore, Mbl-Cre-mediated Drosha [118] and Dicer [118,119] depletion also emphasized the role of miRNAs in the early B-cell lymphopoiesis [117,118,119].

Construction of an atlas of human mature B-cell miRNAs (miRNome) and extensive miRNA array for miRNA profiling in CD5+ and CD5- B-cells together [120,121], elucidated 34 miRNAs enriched in CD5+-activated B-cells [122], of which 8 of them, including miR-323, miR-138, miR-9 *, miR-211, miR-129, miR-373, miR-135a, and miR-184, were highly expressed miRNAs capable of co-targeting ZEB1 and TP53 [123].

To date, mir-181a was found to be highly expressed in the bone marrow, spleen, and thymus, and retroviral expression of mir-181a was confirmed to induce B-cell commitment over other lineages in HSCs, both in vitro and in vivo [113,124]. Although, miR-181-deficient mice models demonstrated a milder disturbed B-cell differentiation [17,18]. Moreover, miR-126 also seems to play a role in B-cell lineage differentiation due to 600-fold higher expression in B-cells in comparison with control cells [125]. Based on the previous evidence, retroviral overexpression of miR-126 in immature B-cells derived from patients with acute lymphoblastic leukemia and mice HSCs was demonstrated to induce B-cell lineage commitment. The underlying mechanism involved the role of miR-126 to negatively regulate insulin regulatory subunit-1 (IRS-1) as a known inhibitor of proliferation and differentiation, while the role of IRS-1 in B-cell commitment remains to be elucidated [125].

The miR-17-92 cluster (including miR-17, 18a, 19a, 20a, 19b-1, and 92a-1) has been reported to be essential for fetal and adult B-cell development [126]. Previous experiments using miR-17-92-deficient mice illustrated the importance of this cluster in the transition of B-cells from the pre- to pro-B-cell stage. The underlying mechanism of miR-17-92 function is regulating Bim as a pro-apoptotic protein; thus, miR-17-92 cluster deficiency leads to death after birth in a mice model [127]. In another experiment, lentiviral overexpression of the miR-17 subfamily in a miR-17-92 knockout mice model was shown to save the conventional B-cell development [128]. Although the described mechanism of miR-17-92 function in the progression of early B-cell development in the latter study is not based on the modulation of Bim, other distinct pathways and/or molecules as targets for the miR-17-92 cluster would be involved [128].

Another miRNA that was proven to be involved in pro- to pre-B-cell transition is miR-34a [129]. Retroviral overexpression of miR-34a was indicated to perturb the B-cell maturation process and arrest of the B-cell transition in the pro- to pre-B-cell stage through repression of Forkhead Box P1 (Foxp1) as a crucial regulator of early B-cell development [129].

Another miRNA with a dynamic expression during all stages of B-cell development is miR-150. Although, the most notable expression of miR-150 was found to be in the immature stage of B-cell lymphopoiesis. The transplantation of mice bone marrow with HSCs overexpressing miR-150 using retroviral vectors was demonstrated to dramatically impair the pro- to pre-B-cell development as well as mature B-cell formation. The mechanism by which miR-150 governs the B-cell development relies on negatively regulating Myb and Foxp1, as indispensable TFs in this process [32,130].

The miR-23a cluster, including miR-23a, miR24-2, and miR-27a, was reported as regulating B-cell lymphopoiesis. The miR-23a cluster is known as a target for the PU.1 transcription factor, which plays a crucial role in immune cell lineages’ differentiation from multipotent progenitors [131]. Generation of the miR-23a cluster-knockout mouse model revealed a considerably increased number of B lymphocytes; however, it occurred at the expense of myeloid cells [132]. Functional in vivo and in vitro studies interestingly demonstrated that overexpression of miR-24-2 alone or along with other miR-23a cluster members could simulate the PU.1 activity, which further induces the differentiation of HSCs into common myeloid progenitors (CMP) [113,133].

In a parallel experiment, overexpression of the miR-23a cluster by retroviral vectors in mouse HSCs blocked the B-cell lineage development in vitro. Moreover, in lethally irradiated animals, B-cell lymphopoiesis was inhibited upon bone marrow transplantation with manipulated HSCs [133]. The possible regulatory mechanism of the miR-23a cluster is targeting c-Myc and H2AX by miR-24-2, leading to the pleiotropic effects on hematopoiesis, including B-cell lymphopoiesis blockade [133].

Another miRNA cluster, the miR-212/132 cluster, is known to have a dynamic role in the regulation of B-cell lymphopoiesis. Transduction of a miR-132-overexpressing vector was shown to arrest the B-cell development in the early stage from pre-B-cell to pro-B-cell in mouse models [134]. On the other hand, B-cell subsets showed no evident decline in miR-132 knockout animal models [135]. The functional analysis revealed that the miR-212/132 cluster exerts an inhibitory effect through the regulation of Sox4 transcription factor [134]. Moreover, a Sox4 knockout mice model showed a systematic reduction in B-cell number, which indicates the role of Sox4 in early B-cell survival. It has been investigated that Sox4 interacts with c-Kit and Bcl-2 to rescue early B-cells from apoptosis [136]. Furthermore, Sox4 was found to be an important TF for the induction of the RAG1 gene, which plays a fundamental role in V(D)J recombination and further differentiation of B-cells [134].

In another confirmatory study, employment of miR-132-overexpressing HSCs for bone marrow reconstitution in a mice model led to inhibition of pro-B-cell differentiation, while differentiation of pro-B-cells was rescued when a Sox4 mutant lacking a miR-132 binding site was applied [108,134].

It can be concluded that miRNAs display positive or negative regulatory effects on B-cell development. MiR-181a, miR-126, and miR-17-92 function as drivers of B-cell lymphopoiesis in bone marrow, while miR-34a, miR-150, miR-23a, and miR-212/132 dramatically regulate B-cell development. All these positive and negative effects of miRNas orchestrate through the interaction of miRNAs with various TFs and mediating complex gene expression signatures. Although, there are definitely more miRNAs with related crosstalk that remain to be defined in B-cell lymphopoiesis.

### 3.2. Periphery

Two distinct types of B-cells are known in the periphery after maturation, including IgM-secreting marginal zone B-cell (MZB), which is important in the early phase of infection [137], and follicular B-cell (FOB). These mature B-cell subsets differentiate from immature B-cells or transitional B-cells upon arrival to the spleen [138].

There are various miRNAs characterized to be involved in marginal and follicular B-cell maturation. Among the differentially expressed miRNAs in B-cell development is miR-146a. Using miR-146a-deficient mice models revealed a profound reduction of splenic MZB cells; however, the transitional T1 and T2 B-cell numbers remained intact. The mechanism of action for miR-146a is direct targeting of Numb protein, which is a repressor of the Notch-2 signaling pathway [139]. Therefore, the increased Numb protein and decreased Notch-2 signaling led to transient B-cell loss of maturation.

On the other hand, miR-142 knockout studies indicated an immunoproliferative response in MZB cells, leading to enlargement in the splenic B-cell compartment, which might be due to targeting the B-cell activating factor receptor (BAFF-R), which leads to the proliferation of MZB in response to BAFF. Subsequently, hypo-immunoglobulinemia was presented in miR-142-deficient animal models, showing a diminished response of B-cells. Moreover, B-cell homeostasis is strictly regulated by miR-142 through the interaction with B-cell-activating factor receptors [140].

Another miRNA with a dynamic role in immune system development and response is miR-155, which can negatively regulate the FOB cell and the B-cell responsiveness to T-cell-dependent and -independent antigens. A miR-155-deficient mice model that presented with a reduced number of FOB cells, while using retroviral overexpression of miR-155, was accompanied by increased numbers of FOB and enhancement of antibody response, concomitantly [74]. Functional analysis indicated the role of miR-155 in negatively regulating activation-induced deaminase (AID) enzyme, which mediates somatic hypermutation and isotype-switching processes in immunoglobulins [141,142]. Thus, it can be concluded that miR-155-deficient B-cells present a reduced follicular response and failure in the secretion of class-switched and high-affinity immunoglobulins [143].

In the final processes of B-cell maturation into plasma cells and further to memory B-cells in the periphery, two major regulatory miRNAs were found: miR-125b, which was observed to inhibit B-cell maturation into plasma cells, and miR-223. In the corresponding study, transfection of lipopolysaccharides-treated B-cells with an miR-125b mimic led to reduced secretion of IgM due to plasma cell maturation arrest. The underlying mechanism of miR-125b function is based upon targeting IRF4 and BLIMP-1 transcription factors, which jointly drive post-germinal center processes, including plasma cell generation and memory B-cell transition [144]. Furthermore, a multiplexed real-time PCR study illustrated the miRNA expression profile in mature B-cells. Among those miRNAs, miR-223 was found to be overexpressed in both naïve and memory B-cells. In this process, miR-223 might target LIM Domain Only 2 and MYB Proto-Oncogene-Like 1 to regulate the transition of FOB into memory B-cells, in addition to naïve cells into FOB cell maturation [121].

**Figure 4:**
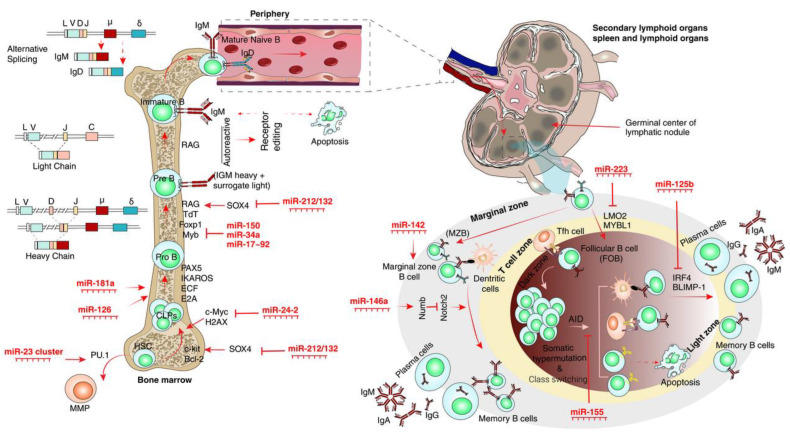
MiRNAs’ roles in B-cell commitment. (**Left**): The maturation process of B-cells from CLP in bone marrow during V(D)J recombination and the role of miRNAs in each step. (**Right**): Role of miRNAs in the differentiation of marginal zone B-cell (MZB) and follicular B-cell (FOB) in peripheral lymphoid organs. Abbreviations: H2AX: H2A histone family member X; TdT: Terminal Deoxynucleotidyl Transferase.

Generally, the in-depth analysis revealed several miRNAs to be implicated in various stages of peripheral B-cell development and function, including the transition to MZB and FOB cells and the generation of plasma and memory B-cells. In this regard, distinct steps such as isotype switching and somatic hypermutation have been targeted by miRNAs. In summary, miR-146a and miR-142 function as enhancers and inhibitors of MZB cell development, respectively. MiR-155 plays a pivotal role in negatively regulating the germinal center maturation and FOB cell responsiveness. MiR-125b and miR-223 were found to be involved as inhibitors of plasma cell maturation and memory B-cell formation, respectively. An overview of the crosstalk of miRNA and TFs is summarized in Supplementary Table S1. The instant findings of B-cell maturation processes are shown in Figure 4.

## 4. Conclusions

The role of epigenetic factors in the regulation of the adaptive immune system during development is incontrovertible. MiRNA, as one of the major epigenetic modulators, plays an important role in driving lymphopoiesis. Some miRNAs were demonstrated to have a major role in the maintenance of stemness in HSCs, e.g., miR-29a, or driving lymphopoiesis, such as miR-125b. One of the most critical miRNAs in T-cell commitment was shown to be miR-181a1/b1, while the leading role for B-cell commitment was assigned to miR-181a and miR-17–92 clusters. Although, the interplay of miRNAs with a web of TFs would eventually orchestrate the immune system’s development. Most of the miRNAs involved in the context of immune system development reveal conflicting roles and functions as effectors or inhibitors, particularly based on the maturity of immune cells. However, there are still more miRNAs and TF interactions yet to be defined in lymphopoiesis. Furthermore, the emergence of exosomal miRNAs has opened new landscapes in the field of our understanding of adaptive immune system evolution. Thus, miRNAs could further be applied as a target for therapy in immunodeficiency and/or autoimmune diseases based on the regulatory potency. Nevertheless, further proof-of-concept studies are still required to establish the usefulness of miRNAs’ application alone or in combination with the conventional target in vitro and in vivo to improve the quality of patients’ lives.

## Data Availability

Not applicable.

## Supplementary Materials

The following supporting information can be downloaded at: https://www.mdpi.com/article/10.3390/cells12040635/s1, Table S1: The crosstalk of miRNA and TFs.
