# Supplementary material for: Crosstalk of Transcriptional Regulators of Adaptive Immune System and microRNAs: An Insight into Differentiation and Development
Source: Cells. 2023 Feb 16;12(4):635. doi: 10.3390/cells12040635 (PMC9953855; doi:10.3390/cells12040635)
Supplement: Supplementary file 1 [file cells-12-00635-s001.zip › cells-2129819-supplementary.pdf]

Table S1. The crosstalk of miRNA and TFs.

	miRNA	Transcription factor	Function	Reference
**T-cell**				
CLP	miR-181a1/b1	Notch-1	Thymocyte development	[17]
CLP	miR-150	Notch-3	Decreasing survival and proliferation of T-cells	[31,32]
CLP	miR-146a	PU.1, GATA3, Runx1	Promoting T-cell proliferation and reducing the number of peripheral CD4+ T-cells	[34,35] [36]
CLP Th2 Treg	miR-155	1.SOCS1, 2.c-Maf 3. Foxp3 4. STAT5	1. Promoting thymocytes proliferation 2. Promoting Th2 cell differentiation 3. Derivation process of Treg from naïve T-cells 4. Modulating thymic generation of Treg cells	[44,45] [72,73] [88,89] [92]
Treg	miR-115, miR-124a	Foxp3	Generating iTreg	[89]
CD4+Th	miR-181a-5p	Cd4 silencer	Governing CD4+ helper cell subset differentiation	[52,53].
Th1	miR-29	Tbx21	Decreasing Th1 cell differentiation	[60]
Th2	miR-126	OBF.1/BOB.1, PU.1, GATA3	Th2 cell differentiation and activation	[50]
Th17	miR-301a	PIAS3	Th17 cell differentiation	[20,50]
Th17	miR-182	STAT5, Foxo1	Th17 differentiation	[43]
Treg	miR-21	STAT3, Ap-1	Inducing homeostasis of Tregs	[82,93]
Tfh	miR-17-92	BCL-6	Positive or a negative regulator of Tfh	[99]
**B-cell**				
	miR-150	Myb, Foxp1	Governing the B-cell development	[32,130]
	miR-23a	PU.1	Increasing number of B lymphocytes	[127]
	miR-24-2 alone or with miR-23a	PU.1	Inducing the differentiation of HSCs into common myeloid progenitors	[109,129]
	miR-212/132	Sox4	B-cell lymphopoiesis	[130]
	miR-34a	Foxp1	Pro-to-pre-B cell transition	[125]
	miR-125b	IRF4, BLIMP-1	Plasma cell generation and memory B-cell transition	[140]
